# SARS-CoV-2 in Egypt: epidemiology, clinical characterization and bioinformatics analysis

**DOI:** 10.1016/j.heliyon.2022.e08864

**Published:** 2022-01-31

**Authors:** Badriyah Alotaibi, Thanaa A. El-Masry, Mohamed G. Seadawy, Mahmoud H. Farghali, Bassem E. El-Harty, Asmaa Saleh, Yasmen F. Mahran, Jackline S. Fahim, Mohamed S. Desoky, Mohamed M.E. Abd El-Monsef, Maisra M. El-Bouseary

**Affiliations:** a Department of Pharmaceutical Sciences, College of Pharmacy, Princess Nourah Bint Abdulrahman University, Riyadh, Saudi Arabia; b Department of Pharmacology and Toxicology, Faculty of Pharmacy, Tanta University, Tanta, Egypt; c Biological Prevention Depart Egypt Army, Egypt; d Department of Pharmaceutical Microbiology, Faculty of Pharmacy, Tanta University, Tanta, Egypt; e Department of Pharmacology & Toxicology, Faculty of Pharmacy, Ain Shams University, Cairo, Egypt; f Researcher at the National Research Center, Egypt; g Department of Biochemistry, Faculty of Pharmacy, Al Azhar University, Cairo, Egypt; h Faculty of Science, Tanta University, Tanta, Egypt

**Keywords:** COVID-19, rRT-PCR, Clinical data, Whole genome sequencing, Haplotypes

## Abstract

COVID-19 is an infectious disease caused by SARS-CoV-2 and has spread globally, resulting in the ongoing coronavirus pandemic. The current study aimed to analyze the clinical and epidemiological features of COVID-19 in Egypt. Oropharyngeal swabs were collected from 197 suspected patients who were admitted to the Army Hospital and confirmation of the positivity was performed by rRT-PCR assay. Whole genomic sequencing was conducted using Illumina iSeq 100® System. The average age of the participants was 48 years, of which 132 (67%) were male. The main clinical symptoms were pneumonia (98%), fever (92%), and dry cough (66%). The results of the laboratory showed that lymphocytopenia (79.2%), decreased levels of haemoglobin (77.7%), increased levels of interleukin 6, C-reactive protein, serum ferritin, and D-dimer (77.2%, 55.3%, 55.3%, and 25.9%, respectively), and leukocytopenia (25.9%) were more common. The CT findings showed that scattered opacities (55.8%) and ground-glass appearance (27.9%) were frequently reported. The recovered validated sequences (n = 144) were submitted to NCBI Virus GenBank. All sequenced viruses have at least 99% identity to Wuhan-Hu-1. All variants were GH clade, B.1 PANGO lineage, and L.GP.YP.HT haplotype. The most predominant subclade was D614G/Q57H/V5F/G823S. Our findings have aided in a deep understanding of COVID-19 evolution and identifying strains with unique mutational patterns in Egypt.

## Highlights

• Isolation and clinical characterization of SARS-CoV-2 from Egyptian patients.
• Whole Genome Sequencing of recovered isolates revealed unique sequences.
• Egyptian SARS-CoV-2 variants in 2020 with at least 99% identity to Wuhan-Hu-1.
• Egyptian SARS-CoV-2 variants were GH clade and L.GP.YP.HT haplotype.
• A unique mutation (D614G/Q57H/V5F/G823S) pattern was predominant among SARS-CoV-2.

## 1 Introduction

The coronavirus disease 2019 (COVID-19) causes a large number of fatal cases worldwide. It is developed as a result of infection with severe acute respiratory syndrome coronavirus 2 (SARS-CoV-2) [1]. This virus is a novel strain related to a bat coronavirus (RaTG13) that has never been isolated in humans [2]. It first appeared in Wuhan city, China in early December 2019, and owing to its tremendous rapid expansion and fatal cases, the World Health Organization (WHO) considered it ‘Public Health Emergency of International Concern’ [2, 3]. As of 18^th^ Nov. 2021, globally, 254,847,065 COVID-19 cases and 5,120,712 deaths have been reported to WHO, while 346,808 cases and 19,707 deaths have been reported from Egypt (https://covid19.who.int/region/emro/country/eg). SARS-CoV-2, one of the coronaviruses family, is blamed for its widespread infection reaching more than 4 million people, a property that characterizes SARS-CoV-2from the other coronaviruses' infections. It was documented that during medical procedures, the transmission of SARS-CoV-2 can be either by droplet or direct/indirect contact. There are several signs and symptoms of SARS-CoV-2 infection, such as flu-like symptoms, fever, cough, difficulty of breathing, fatigue, and loss of smell and taste [2].

Indeed, coronavirus sequences are undergoing continuous changes because of mutation and recombination [4]. In this context, it was suggested that phylogenetic analysis of SARS-CoV-2 genomes might track down the path of transmission within a hospital, recognize the source of the outbreak and help in infection prevention and control strategies [5]. Although studies found similarity in the genome sequence of SARS-CoV-2 and the earlier reported SARS-CoV and Middle East respiratory syndrome coronavirus (MERS-CoV), drugs that show efficacy against both SARS-CoV and MERS-CoV have not shown any efficiency in managing SARS-CoV-2 [6]. Accordingly, understanding the pattern of changes in the future and how the path of the virus will be is becoming necessary.

According to the phylogenetic analysis classification, all coronaviruses are enveloped viruses and belong to Baltimore class IV viruses (+ssRNA). Moreover, 7 species of human coronaviruses have been reported: two species are belonging to the Alpha genus; HCoV-NL63 and HCoV-229E, and five species are belonging to the Beta genus; HCoV-OC43, HCoVHKU1, SARS-CoV, MERS-CoV, and SARS-CoV-2. Consequently, SARS-CoV-2 is classified as β-Coronavirus which consists of 4 structural proteins; nucleocapsid protein (NP), spike protein (S), envelope protein (E), and membrane protein (M). The membrane protein is responsible for constructing the viral envelope which has spikes on its surface providing the virus its unique crown-like appearance [7, 8]. During infection, the template mRNA of NP is the most common sub-genomic RNA that is formed and shed. As being highly immunogenic, it is detected in either serum or urine during early infection (approximately the first two weeks) [9]. Furthermore, the spike protein is a glycoprotein in nature and has two subunits; S1 and S2, and it is known as the main target for neutralizing antibodies and vaccines. In this context, S1 contains the receptor-binding domain (RBD) that recognizes and binds with the host cell receptor and the most variable immunogenic antigen [8, 10].

Besides, the real-time reverse transcription-polymerase chain reaction (rRT-PCR) is one of the best, precise and reliable in vitro diagnostic assays for confirmatory diagnosing and studying the causative coronavirus from respiratory secretion. Real-time RT-PCR has been employed worldwide to detect SARS-CoV-2 due to minimal chances of false-positive results [11, 12]. Real-time RT-PCR was designed to detect ORF1b gene and N regions of SARS-CoV-2 in less than 3h including the RNA extraction from the samples [12].

Reviewing the literature, previous studies conducted in Egypt focused on either the investigation of ongoing mutations or on the clinical characterization of COVID-19. In this study, we isolate SARS-CoV-2 from oropharyngeal swabs and perform next-generation sequencing (NGS) which is of great importance in identifying different SARS-CoV-2 mutations. Furthermore, we combine patient clinical data and in-depth genomics to help in a deep understanding of the evolution of SARS-CoV-2.

## 2 Results

### 2.1 Description of the study population and clinical parameters

**Figure 1:**
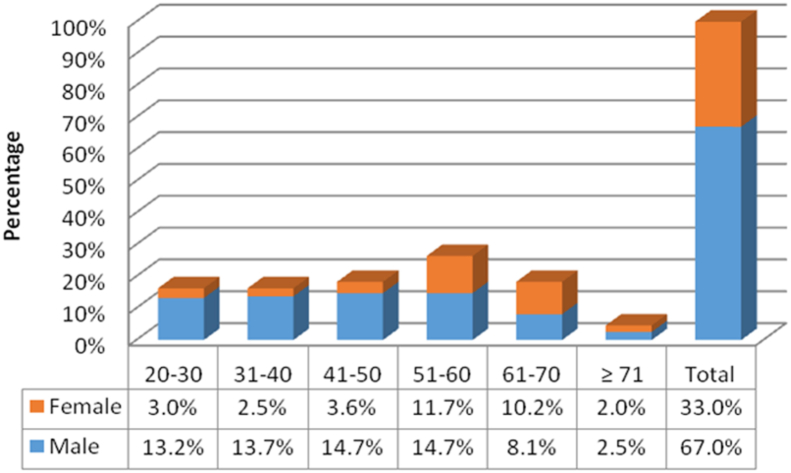
Bar chart showing the percentage of female and male distribution among different age groups.

A total of 197 clinically diagnosed positive COVID-19 patients admitted to the Army hospital from April 2020 to December 2020 were included in the present work, 132 (67%) males and 65 (33%) females with an approximate mean age of 48 and their ages ranged from 20-75 years (Figure 1).

The distribution of participants among months of admittance and the duration of hospitalization are presented in Table 1. The highest percentage of cases was recorded in May (n = 81, 41%) with a gradual decrease in June (n = 25, 12.7%) and July (n = 4, 2%), followed by a constant low percent in August and September (1%). The percent increased again in October (n = 5, 2.5%) and continued in rising in November and December (7.6% and 13.7%, respectively). All participants required hospitalization for variable periods of time, so, we categorized patients into four groups according to the duration of hospitalization, the first group for ˂ 14 days (21%), the second group for ≥14–21 days (26%), the third group for ≥22–28 days (20%) and the last one for ≥29 days (28%). Ten participants died after the collection of samples with an overall death rate of 5%.

**Table 1:** The distribution of participants among months of admittance and the duration of hospitalization.

Month		Apr	May	Jun	Jul	Aug	Sep	Oct	Nov	Dec	Total
Hospitalization ˂ 14 days	No.	11	13	2	0	0	0	3	7	6	**42**
	%	26.2%	31.0%	4.8%	0.0%	0.0%	0.0%	7.1%	16.7%	14.3%	**100%**
Hospitalization ≥14–21 days	No.	3	22	4	0	0	0	1	6	9	**45**
	%	6.7%	48.9%	8.9%	0.0%	0.0%	0.0%	2.2%	13.3%	20.0%	**100%**
Hospitalization ≥22–28 days	No.	9	19	5	0	0	1	0	2	3	**39**
	%	23.1%	48.7%	12.8%	0.0%	0.0%	2.6%	0.0%	5.1%	7.7%	**100%**
Hospitalization ≥29 days	No.	10	18	11	4	2	1	1	0	8	**55**
	%	18.2%	32.7%	20.0%	7.3%	3.6%	1.8%	1.8%	0.0%	14.5%	**100%**
Death	No.	3	9	3	0	0	0	0	0	1	**16**
	%	18.8%	56.3%	18.8%	0.0%	0.0%	0.0%	0.0%	0.0%	6.3%	**100%**
Total	No.	**36**	**81**	**25**	**4**	**2**	**2**	**5**	**15**	**27**	**197**
	%	**18.3%**	**41.1%**	**12.7%**	**2.0%**	**1.0%**	**1.0%**	**2.5%**	**7.6%**	**13.7%**	**100%**

The clinical symptoms of the patients enrolled in the current study are shown in Table 2. Pneumonia and fever were the most common symptoms among the patients (98% and 92%, respectively), followed by dry cough (66%) and dyspnea (21%). Only three patients (2%) suffered from sore throats.

**Table 2:** Clinical symptoms of SARS-CoV-2 patients involved in the current study.

Clinical Symptoms	No.(%) of patients showing symptoms according to duration			
	0 day	1–7 days	8–14 days	>14 days
Fever	16 (8.1%)	135 (68.5%)	23 (11.7%)	23 (11.7%)
Sore throat	194 (98.5%)	2 (1.0%)	1 (0.5%)	0 (0.0%)
Dyspnea	155 (78.7%)	22 (11.2%)	5 (2.5%)	15 (7.6%)
Dry cough	67 (34.0%)	90 (45.7%)	14 (7.1%)	26 (13.2%)
Pneumonia	4 (2.0%)	54 (27.4%)	79 (40.1%)	60 (30.5%)

Laboratory test results obtained at the time of patient admission and recorded three more consecutive times with an interval of 4–5 days to follow up on disease progression are presented in Table 3.

**Table 3:** Laboratory findings obtained at the time of patient admission.

Variable	Reference range		Lab test repeat	Normal range frequency (%)	Abnormal range frequency (%)	Chi-Square test P value	Median	∗IQR	P value
HB^1^	Male Female	13–17 g/dL 12–16 g/dL	HGB 1	44 (22.3)	153 (77.7)	˂0.001	13.50	2.86	<0.001
			HGB 2	95 (48.2)	102 (51.8)	˂0.001	14.50	0.00	<0.001
			HGB 3	170 (86.3)	27 (13.7)	0.196	15.56	0.00	<0.001
			HGB 4	188 (95.4)	9 (4.6)	0.478	15.56	0.00	<0.001
			HGB 5	194 (98.5)	3 (1.5)	0.686	15.56	1.25	<0.001
Lymph^2^	1–3 × 10^3^/μL		Lymph 1	41 (20.8)	156 (79.2)	˂0.001	1.62	0.94	<0.001
			Lymph 2	6 (30.5)	137 (69.5)	0.037	2.56	0.00	0.065
			Lymph 3	185 (93.9)	12 (6.1)	0.408	2.56	0.00	<0.001
			Lymph 4	195 (99)	2 (1)	0.742	2.56	2.07	0.999
Neut^3^	2–7 × 10^3^/μL		Neut 1	155 (78.7)	42 (21.3)	0.370	3.31	2.23	<0.001
			Neut 2	162 (82.2)	35 (17.8)	0.510	2.56	0.00	0.003
			Neut 3	185 (93.9)	12 (6.1)	0.408	2.56	0.00	0.003
			Neut 4	191 (97)	6 (3)	0.565	2.56	0.00	0.003
PT^4^	12–16 s		PT 1	194 (98.5)	3 (1.5)	0.686	15.56	0.00	<0.001
			PT 2	196 (99.5)	1 (0.5)	0.817	15.56	0.00	<0.001
			PT 3	196 (99.5)	1 (0.5)	0.817	15.56	0.00	<0.001
			PT 4	196 (99.5)	1 (0.5)	0.817	15.56	0.00	<0.001
CRP^5^	˂6 mg/L		CRP 1	88 (44.7)	109 (55.3)	0.021	5.56	0.00	<0.001
			CRP 2	196 (99.5)	1 (0.5)	0.817	5.56	0.00	<0.001
			CRP 3	196 (99.5)	1 (0.5)	0.817	5.56	0.00	<0.001
			CRP 4	197 (100)	- (0)	**a**	5.56	0.00	<0.001
INR^6^	1		INR 1	176 (89.3)	21 (10.7)	0.326	1.00	0.00	<0.001
			INR 2	192 (97.5)	5 (2.5)	0.600	1.00	0.00	<0.001
			INR 3	195 (99)	2 (1)	0.742	1.00	0.00	<0.001
			INR 4	195 (99)	2 (1)	0.742	1.00	34.80	<0.001
IL6^7^	0–16.4 pg/ml		IL 6 1	45 (22.8)	152 (77.2)	0.006	16.64	48.03	<0.001
			IL 6 2	72 (36.5)	125 (63.5)	0.024	15.56	17.09	<0.001
			IL 6 3	86 (43.7)	111 (56.3)	0.017	15.56	46.16	<0.001
			IL 6 4	146 (74.1)	51 (25.9)	0.239	15.56	0.00	<0.001
			IL 6 5	162 (82.2)	35 (17.8)	0.510	15.56	0.00	<0.001
			IL 6 6	162 (82.2)	35 (17.8)	0.510	15.56	0.00	<0.001
S- ferritin	Male Female	20–250 ng/ml 10–120 ng/ml	S- fer 1	88 (44.7)	109 (55.3)	0.021	55.56	0.00	<0.001
			S- fer 2	192 (97.5)	5 (2.5)	0.565	55.56	0.00	<0.001
			S- fer 3	196 (99.5)	1 (0.5)	0.817	55.56	0.00	<0.001
			S- fer 4	197 (100)	- (0)	**a**	55.56	0.06	<0.001
D- dimer	˂0.5 μg/mL		D-dimer 1	111 (56.3)	86 (43.7)	0.028	0.06	0.00	<0.001
			D-dimer 2	194 (98.5)	3 (1.5)	0.686	0.06	0.00	<0.001
			D-dimer 3	193 (98)	4 (2)	0.640	0.06	0.00	<0.001
			D-dimer 4	197 (100)	- (0)	**a**	0.06	25.00	<0.001
ALT^8^	7–56 IU/L		ALT 1	173 (87.8)	24 (12.2)	0.438	29.00	20.56	<0.001
			ALT 2	171 (86.8)	26 (13.2)	0.206	55.56	0.00	<0.001
			ALT 3	183 (92.9)	14 (7.1)	0.369	55.56	0.00	<0.001
			ALT 4	194 (98.5)	3 (1.5)	0.686	55.56	20.00	<0.001
AST^9^	5–40 IU/L		AST 1	156 (79.2)	41 (20.8)	0.387	28.00	0.00	<0.001
			AST 2	194 (98.5)	3 (1.5)	0.686	5.56	0.00	<0.001
			AST 3	197 (100)	- (0)	**a**	5.56	0.00	<0.001
			AST 4	197 (100)	- (0)	**a**	5.56	0.44	<0.001
Creat^10^	Male Female	0.7–1.3 mg/dL 0.6–1.1 mg/dL	Creat 1	39 (19.8)	158 (80.2)	0.014	0.90	0.24	<0.001
			Creat 2	42 (21.3)	155 (78.7)	0.023	0.76	0.00	<0.001
			Creat 3	189 (95.5)	8 (4.1)	0.504	0.76	0.00	<0.001
			Creat 4	194 (98.5)	3 (1.5)	0.686	0.76	16.50	<0.001
Urea	20–40 mg/dL		Urea 1	131 (66.5)	66 (33.5)	0.810	29.00	3.44	<0.001
			Urea 2	153 (77.7)	44 (22.3)	0.550	25.56	0.00	<0.001
			Urea 3	180 (91.4)	17 (8.6)	0.319	25.56	0.00	<0.001
			Urea 4	191 (97)	6 (3)	0.565	25.56	0.00	<0.001
LDH^11^	140–280 U/L		LDH 1	184 (93.4)	13 (6.6)	0.388	155.56	0.00	<0.001
			LDH 2	196 (99.5)	1 (0.5)	0.817	155.56	0.00	<0.001
			LDH 3	197 (100)	- (0)	**a**	155.56	0.00	<0.001
			LDH 4	197 (100)	- (0)	**a**	155.56	0.00	<0.001

**∗** IQR; Interquartile range, **1**; Haemoglobin, **2**; Lymphocytes, **3**; Neutrophils, **4**; Prothrombin time, **5**; C-reactive protein, **6**; International normalized ratios, **7**; Interleukin 6, **8**; Alanine aminotransferase, **9**; Aspartate aminotransferase, **10**; Creatinine, **11**; Lactate dehydrogenase and **a;** No statistics are computed.

Complete blood picture (CBC) revealed that the haemoglobin (HB) values were low in 77.7%, 51.8% followed by 13.7%, and finally 4.6% of patients, indicating anaemia. While the low level of white blood cell (WBC), leucopenia, was reported in 25.9% of patients at the time of admission followed by 16.8% in the second test. Either neutropenia or neutrophilia was detected in 21.3% of patients at the time of admission. The percentage of patients showing lymphopenia (the low level of lymphocytes) was 79.2%, then 69.5%, followed by 6.1%, and finally 1.0%. Moreover, the decrease in platelets count, thrombocytopenia was recorded in 15% of the patients at admission time. The patients' complete blood picture showed an improvement in outcomes over time, due to the medical care the patients received. This was demonstrated by the statistical analysis of the results, which showed that both anemia and lymphopenia were significant at the time of admission (P < 0.001) and then switched to non-significant at the last repetition.

The levels of alanine aminotransferase (ALT), aspartate aminotransferase (AST), D-dimer, and lactate dehydrogenase (LDH) were elevated in 12.2%, 20.8%, 43.7%, and 6.6% of patients, respectively. The percentage of patients showed an increase of both C-reactive protein (CRP) and ferritin was 55.5%. However, prothrombin ratios (PT) and international normalized ratios (INR) were normal in 98.5% and 89.3% of patients, respectively.

### 2.2 Confirmation of the positivity of the samples using rRT-PCR assay

Oropharyngeal swabs were collected from all clinically diagnosed positive COVID-19 patients. The samples were tested using Real-Time Polymerase Chain Reaction (RT-PCR) for rough semi-quantitative comparison of viral loads. One hundred and eighty-one samples were positive confirming the infection based on molecular genetics technique.

**Figure 2:**
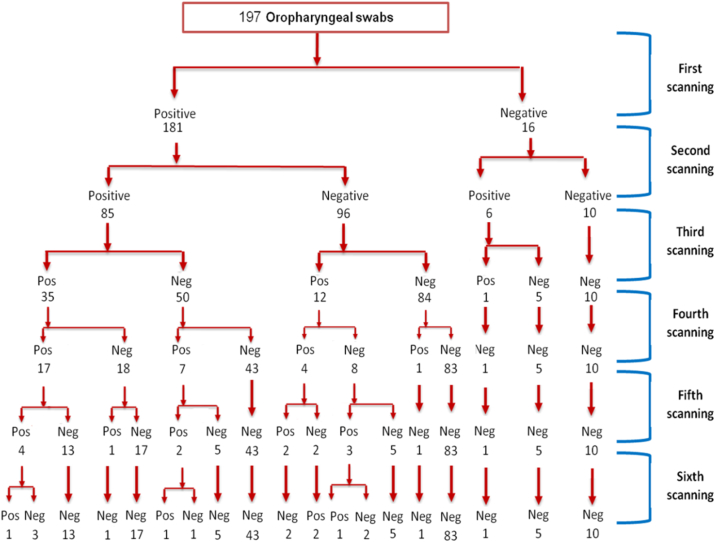
Flow chart showing the results of successive rRT-PCR tests.

For further confirmation, other swabs were collected from all patients after 4–5 days to exclude any false-negative results from the first scanning and also to follow up on the progress of infections. Six out of 16 negative samples from the first scanning were positive in the second one. Moreover, 96 out of 181 positive samples from the first run were negative in the second run. Following up the progress of the disease was continued by the collection of several swabs from each patient under investigation at the interval time of 4–5 days. Regarding the results of the third and fourth runs, 48 and 29 samples were positive in the third and fourth tests, respectively. The results of rRT-PCR assay were presented in a flow chart (Figure 2).

### 2.3 Whole genome sequencing of the isolated strains

Whole genome sequencing (WGS) was performed on 184 isolated strains. The list of validated sequences (n = 144), their IDs in the present study and their accession codes in NCBI virus GenBank database was presented in Supplementary File S1. All variants were GH clade and fall in the B.1 lineages.

**Figure 3:**
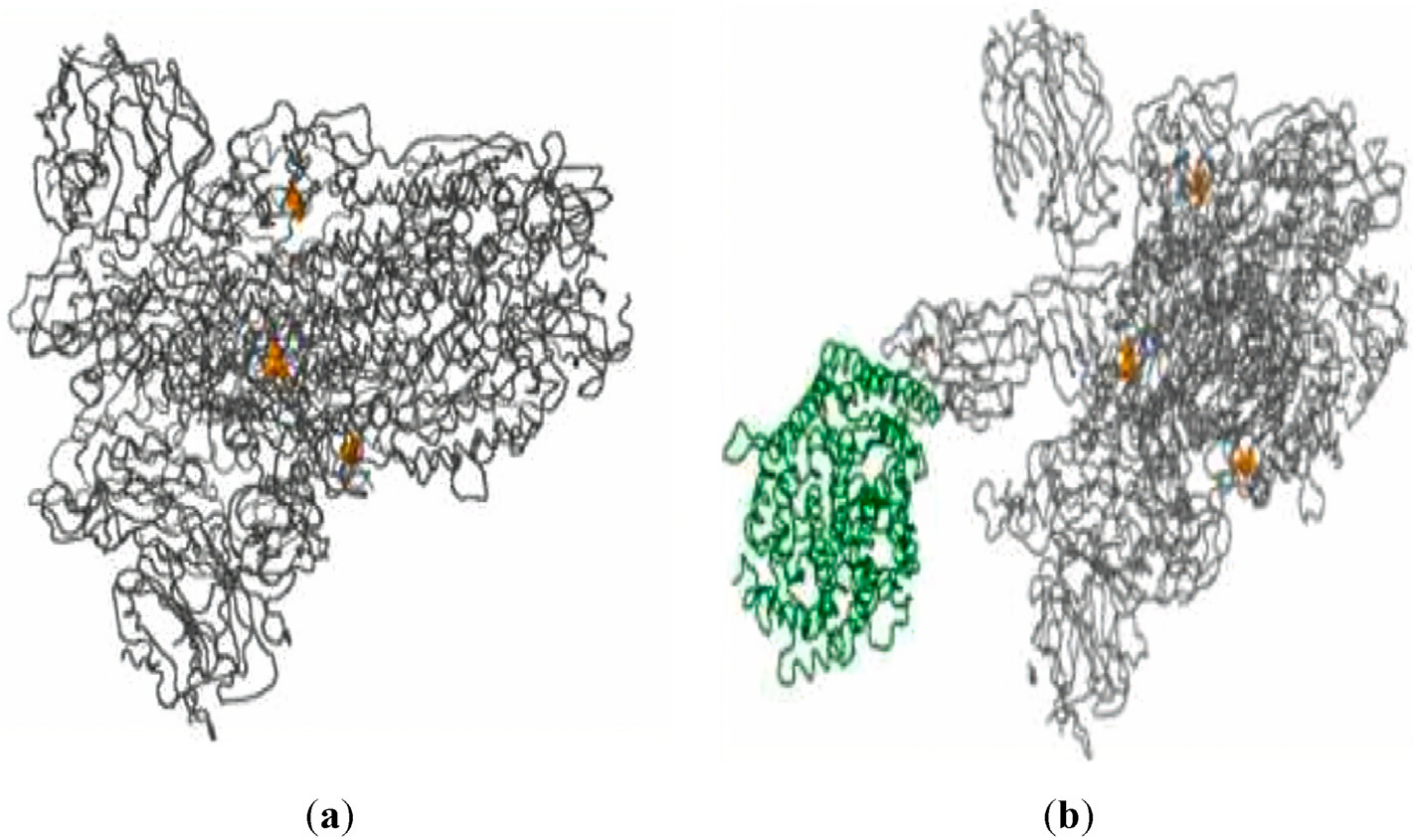
3D structural visualization of the spike glycoprotein with amino acid changes identified in the query sequences shown as colored balls, (a) Spike glycoprotein (PDB: 6acc, EM 3.6 Å) with RBD in down conformation. (b) Spike glycoprotein (PDB: 6acj, EM 4.2 Å) in complex with host cell receptor ACE2 (green ribbon). https://www.gisaid.org/epiflu-applications/covsurver-mutations-app/.

Moreover, all sequences were stored in GISAID, CoVsurver Mutation Analysis of hCoV-19 (https://www.gisaid.org/epiflu-applications/covsurver-mutations-app/), for detection of possible mutations. CoVsurver results for one query were recorded as a representative example for the rest of sequences (see Supplementary File S3). All imported sequences showed the same variations displayed in the structure of the spike glycoprotein with amino acid changes identified as colored balls (Figure 3). The percent of amino acids (AA) identity with respect to the NCBI reference strain, Wuhan-Hu-1 (Accession NC_045512), was 99.92%. Four mutations detected together in all 144 sequences were E_V5F, Spike_D614G, NS3_Q57H, and NSP12_G823S.

### 2.4 Phylogenetic analysis

**Figure 4:**
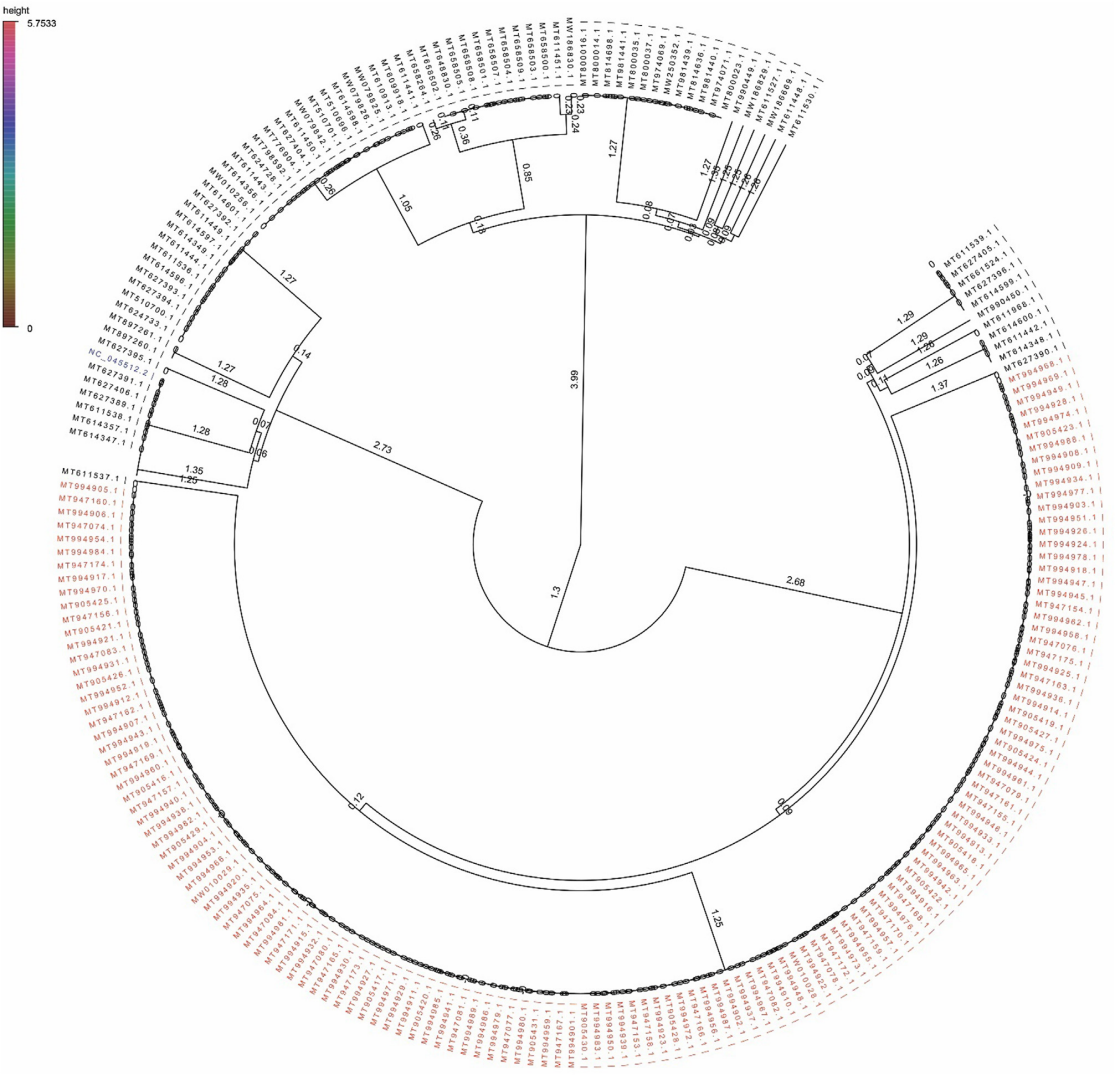
Maximum clade credibility tree constructed using the SARS CoV-2 genome of 240 Egyptian variants collected in 2020. The variants isolated in the current study (n = 144) are labeled red and reference Wuhan-Hu-1 is labeled blue.

The maximum clade credibility (MCC) tree was constructed using a total of 241 sequences including 144 sequences of variants involved in the present study, Wuhan-Hu-1 (Accession NC_045512), and 96 sequences of Egyptian variants released in 2020 (Figure 4). The phylogenetic study of the full sequences revealed that all investigated variants (n = 144) are closely related to NCBI reference strain, Wuhan-Hu-1.

### 2.5 Bioinformatics analysis

As shown in the methodology section, we extracted 27579 SARS-CoV-2 genomic sequences released in 2020 and originating from Egypt (n = 240), Tunisia (n = 43), Morocco (n = 10), Saudi Arabia (n = 58), China (n = 239), India (n = 701), Italy (n = 104), France (n = 89), Greece (n = 98), Germany (n = 93) and USA (n = 25904) from the NCBI Genbank database. Countries involved in the bioinformatics analysis were chosen based on the geographical location including Mediterranean countries such as Tunisia, Morocco, Italy, France & Greece as well as, neighboring countries such as Saudi Arabia and also, countries that are characterized by a continuous movement of travelers between them and Egypt such as USA, Germany, China & India.

### 2.6 Clustering SNP data into dendrograms

**Figure 5:**
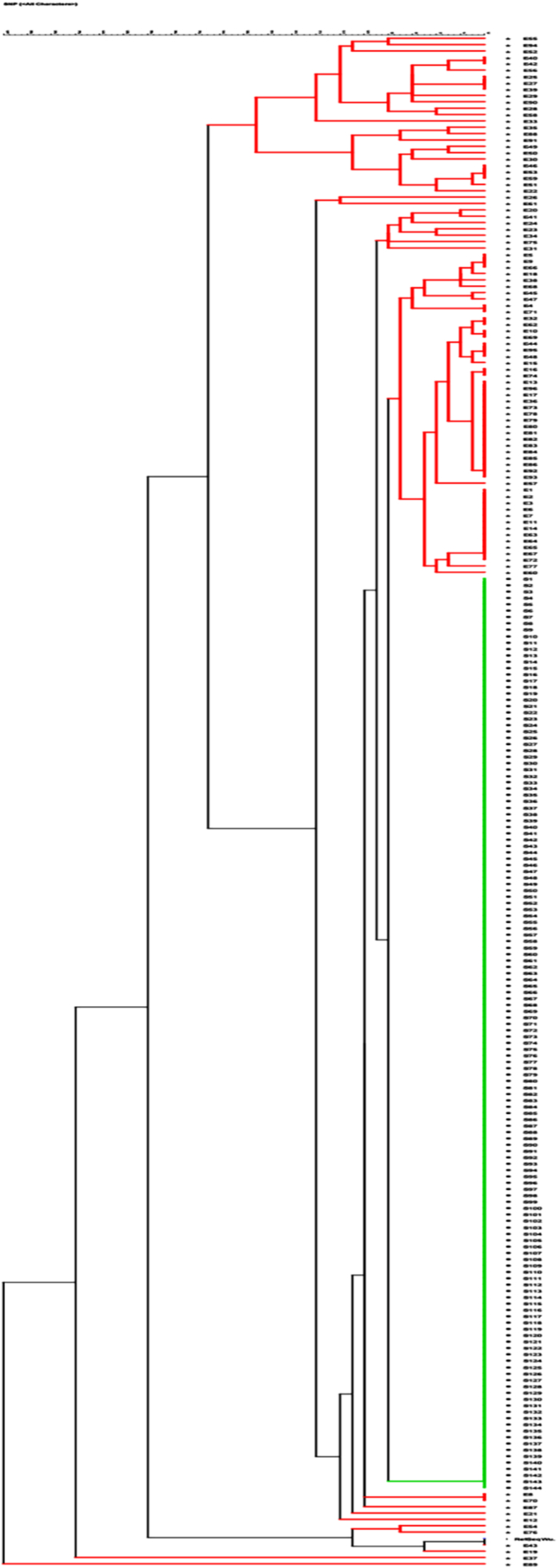
A dendrogram for Egyptian isolates (n = 240). The variants isolated in the present study (n = 144) are labeled green and other Egyptian variants (n = 96) are labeled red (A high-resolution plot is presented in Supplementary File S4).

As shown in the methodology section, after filtering the entries, a total of 18049 sequences were ready for clustering into dendrograms. These sequences were originating from Egypt (n = 235), Tunisia (n = 34), Morocco (n = 10), Saudi Arabia (n = 57), China (n = 623), India (n = 143), Italy (n = 51), France (n = 86), Greece (n = 95), Germany (n = 92) and USA (n = 16649) (see Supplementary File S2). A dendrogram was constructed based on the complete linkage/furthest neighbor clustering algorithm for all Egyptian isolates (n = 240) (Figure 5).

### 2.7 Haplotype determination

**Figure 6:**
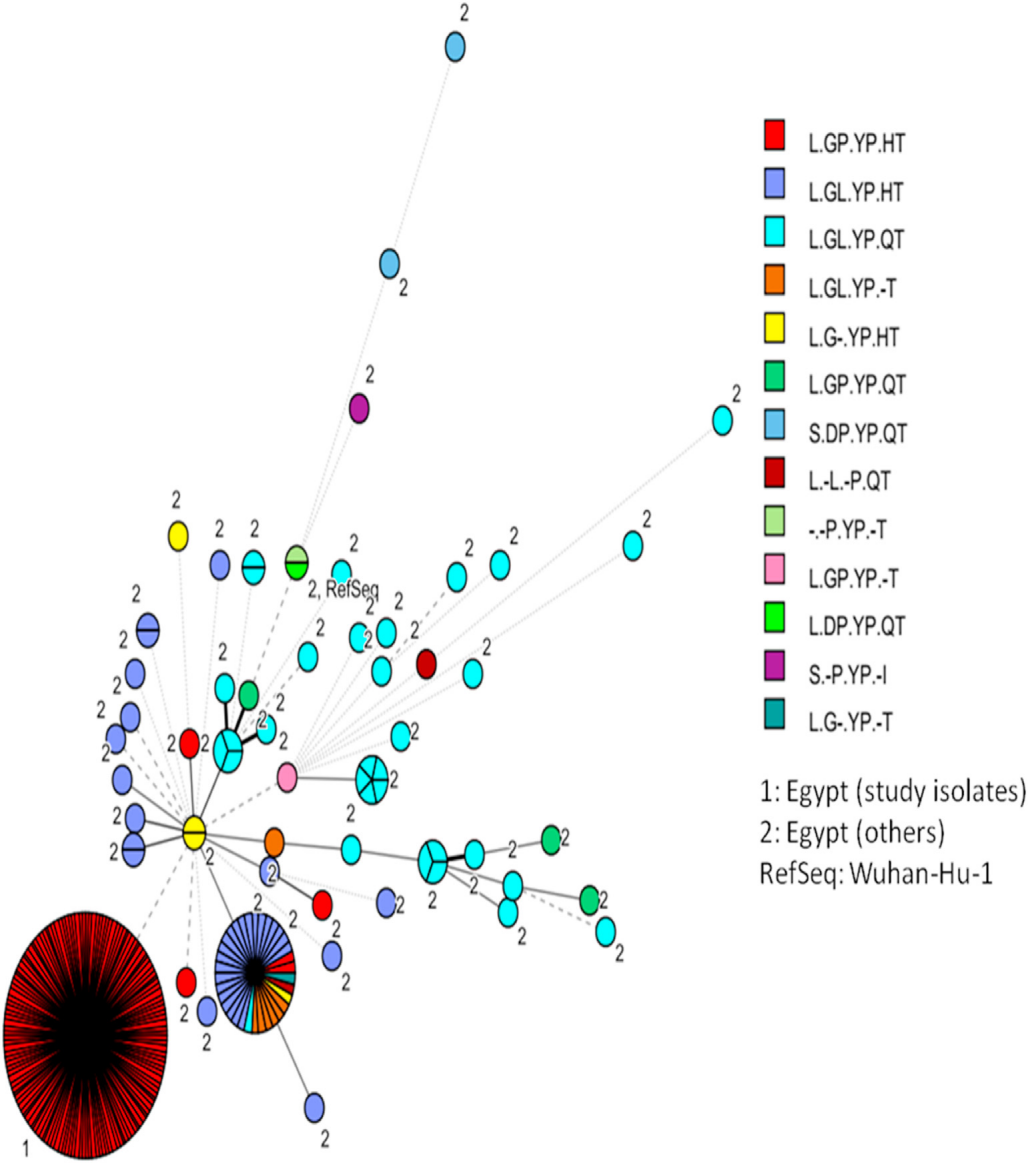
Minimum spanning tree (MST) for Egyptian SARS-CoV-2 genomes (n = 240) included in the current study. Genomes are color-coded by haplotype and labeled by country.

**Figure 7:**
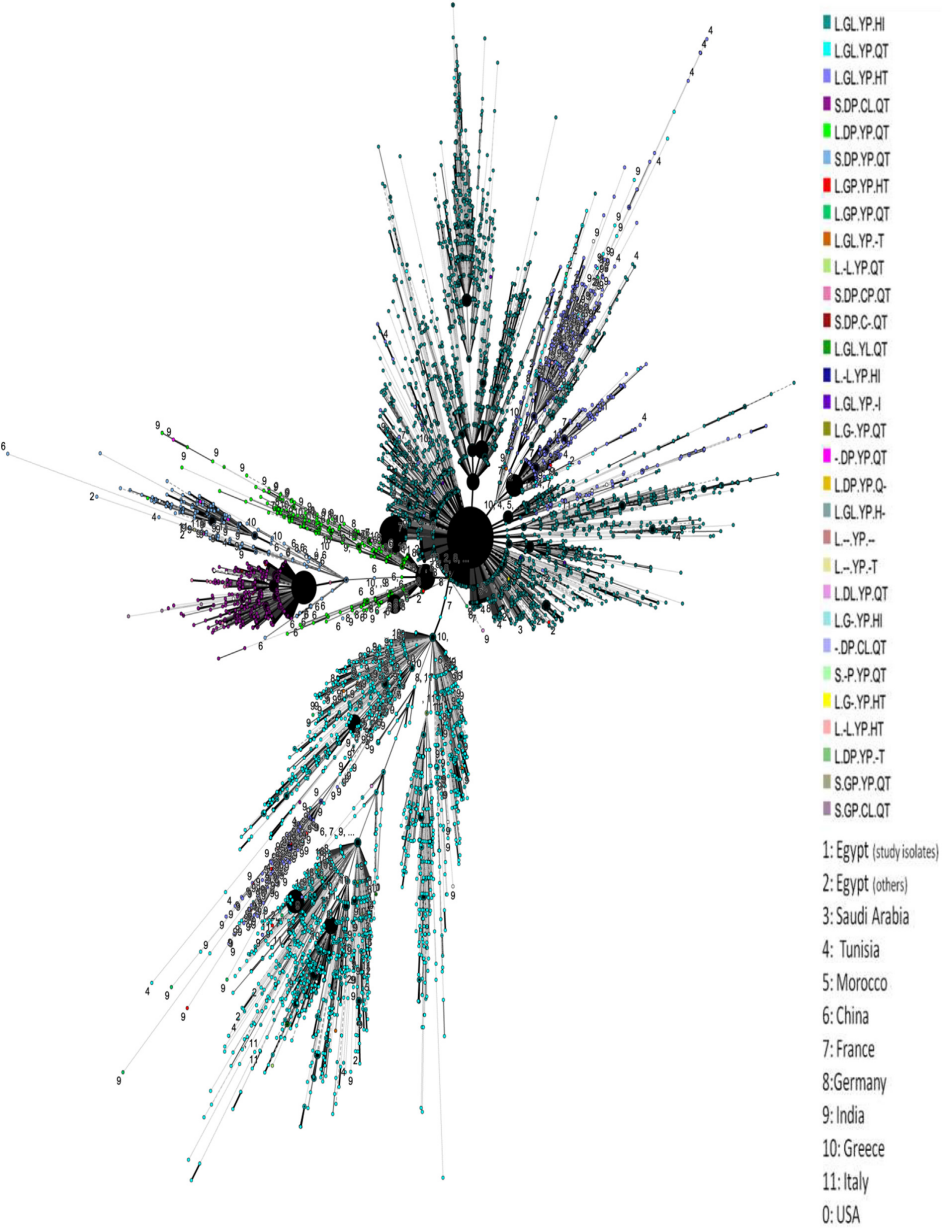
Minimum spanning tree (MST) for all SARS-CoV-2 genomes (n = 18049) included in the present study. Genomes are color-coded by haplotype and labeled by country. (A high-resolution plot is presented in Supplementary File S5).

The haplotypes were determined for each isolate using the built-in BIONUMERICS "Get haplotypes" tool (see Supplementary File S1 & S2). A minimum spanning tree (MST) for all Egyptian SARS-CoV-2 genomes was calculated by BIONUMERICS, using default priority rule settings (Figure 6). It was also constructed for all imported SARS-CoV-2 genomes (n = 18049) (Figure 7).

The distribution of each haplotype in different countries was determined (Table 4). The most frequent haplotypes were L.GL.YP.HI (n = 7954), L.GL.YP.QT (n = 5044), L.GL.YP.HT (n = 1869), S.DP.CL.QT (n = 1490), L.DP.YP.QT (n = 787), S.DP.YP.QT (n = 467) and L.GP.YP.HT (n = 173). Total of 142 Egyptian SARS-CoV-2 isolates recovered in the present study were L.GP.YP.HT, representing 83.24% of the former haplotype recovered in countries under investigation in 2020. The haplotype L.GP.YP.HT was also recovered from India (n = 23), Egypt (n = 7) and France (n = 1).

**Table 4:** The incidence and geographic distribution of SARS-CoV-2 haplotypes.

Haplotype	USA	IND∗	EGY∗	GER∗	FRA∗	GRE∗	KSA∗	ITA∗	TUN∗	MOR∗	CHI∗	Total
L.GL.YP.HI	7887	2		28	19	2	2		9	5		7954
L.GL.YP.QT	4595	222	30	25	32	68	23	29	11	4	5	5044
L.GL.YP.HT	1474	299	37		10	5	30	2	11	1		1869
S.DP.CL.QT	1490											1490
L.DP.YP.QT	631	28		21	19	14	1	2			71	787
S.DP.YP.QT	390	13	2	1		3	1	13	3		41	467
L.GP.YP.HT		23	149		1							173
L.GP.YP.QT	4	9	3	15	4	1						36
L.GL.YP.-T	5	7	7					3				22
L.-L.YP.QT	14	1						2				17
S.DP.CP.QT	16											16
S.DP.C-.QT	15											15
L.GL.YL.QT	13											13
L.-L.YP.HI	11											11
L.GL.YP.-I	9											9
-.DP.YP.QT	4	4										8
L.G-.YP.QT	8											8
L.DP.YP.Q-	5					2						7
L.GL.YP.H-	6											6

∗ IND; India, EGY; Egypt, GER; Germany, FRA; France, GRE; Greece, KSA; Saudi Arabia, ITA; Italy, TUN; Tunisia, MOR; Morocco and CHI; China.

The predominant haplotype in each of the countries under investigation was (L.GL.YP.HI) in USA, Germany, and Morocco, (L.GL.YP.HT) in India, (L.GP.YP.HT) in Egypt, (L.GL.YP.QT) in France and Greece, (L.GL.YP.HT) in Saudi Arabia, (L.GL.YP.QT) in Italy (both L.GL.YP.QT and L.GL.YP.HT), in Tunisia, and (L.DP.YP.QT) in China.

## 3 Discussion

In the present study, we described the epidemiology, clinical characteristics, and laboratory parameters of 197 patients suspected to be COVID-19 positive and showed possible symptoms of infection. We observed that SARS-CoV-2 infections were frequent in the middle-aged group. This is in agreement with previous studies reporting that the group of 40–60 years were the most commonly infected one [13, 14, 15, 16, 17]. Moreover, we reported a higher percentage of infection among males than females. This observation is similar to the findings of other studies [16, 18]. The high incidence of COVID-19 in males could be attributed to sex-based immunological differences that contribute to differences in the susceptibility to contagious diseases [18, 19].

The first COVID-19 case was registered in Egypt on February 14, 2020 [20]. We recorded the highest percentage of cases in May and this was in agreement with previous reports regarding the daily number of COVID-19 cases in Egypt [20, 21]. The former observation showed the effectiveness of the lockdown strategy to control the COVID-19 spread until May when a relaxation of lockdown measurement in Ramadan and Festival break led to an increase in the number of cases and then followed by a decrease in the cases number due to continuous awareness of the seriousness of the disease, its modes of transmission and the emphasis on the need to follow protection measures to reduce the risk of infection.

In the current study, all participants were hospitalized, and the length of stay (LOS) was categorized. Most patients required hospitalization for more than 14 days. Consistent with these findings are the results of the reports that have also recorded that the median LOS of COVID-19 patients ranged from 5 to 29 days [22, 23, 24]. Concerning the clinical symptoms associated with COVID-19 infection, pneumonia, fever, dry cough, and dyspnea were the most frequent symptoms. A chi-square test was applied with *P-value* = 0.000 (highly significant). The former finding is consistent with several previous studies [17, 25, 26, 27, 28]. Until now, the complete clinical picture of COVID-19 infection is not yet clear, and its clinical manifestations are variable and range from asymptomatic to severe cases [25].

In the present study, our laboratory tests were CBC, AST, ALT, LDH, CRP, D-dimer, INR, and IL6. In the beginning, the patients showed low levels of haemoglobin (77.7% of the patients), then improved through the clinical course of the disease to be recorded in only 4.6% of the patients who still had anaemia during convalescence despite high ferritin levels (55.3% of the patients). This observation suggested that most cases of anemia were due to inflammation. Also, there is a relation between the severity of infection and the outcomes of anaemia. This is consistent with the haemoglobin findings in another research [29, 30, 31]. Either neutropenia alone or both leucopenia and neutrophilia were observed in some cases while the majority suffered from lymphopenia. Liu *et al.* stated that neutrophilic and monocytic counts are usually within the normal range but neutrophilia indicates the severity of the disease and most of the patients were ICU ones [32]. While Huang *et al.* observed leucocytosis and lymphopenia in COVID-19 patients and had no explanation for lymphopenia. It has been hypothesized that this correlation could be a consequence of direct lymphocyte infection, damage of lymphatic tissue, inflammation resulting in apoptosis of lymphocytes, or suppression of lymphocytes by metabolic defects. Lymphopenia as a marker of severity has been used to predict viral pneumonia, such as in influenza infection, and so it is not specific to COVID-19. Huang *et al.* also stated that neutrophilia may be more associated with disease severity than leukocytosis [33]. Our results agreed with the others that thrombocytopenia in COVID-19 patients is associated with increased severity of COVID-19 pneumonia [33, 34].

Increased levels of CRP and ferritin, despite normal haemoglobin levels in patients, are markers of inflammation and correlated with the diameter of lung lesions and severe presentation. While elevated levels of enzymes ASL, ALT, and LDH may indicate the level of organ harm in systemic diseases and may serve as biological markers for the progress of COVID-19 infection. In critically ill patients, IL6 is increased as a marker of inflammation [17, 35, 36]. Although, Statsenko *et al.* stated that there is evidence that elevated IL-6 levels do not indicate the level of COVID-19 progression [36].

The elevated D-dimer levels commonly recorded in the patients (43.7% of patients) are associated with a worse prognosis as increased incidence of pulmonary embolism in COVID-19. In this condition, the D-dimer concentration will rise because it is a product of the degradation of a blood clot formed out of fibrin protein. Thromboembolic complications explain the association of low levels of platelets, increased levels of D-dimer, and increasing levels of prothrombin in COVID-19. Many researchers are agreed with us [17, 25, 36]. Both PT and INR levels were almost normal in COVID-19 patients and the little increase in some of them may be related to the received therapy.

Regarding Computed tomography (CT), chest findings such as ground glass appearance indicate the partial filling of the lung alveoli by fluid, interstitial thickening, or partial collapse of lung alveoli. Other findings include scattered opacities and lower lobe pneumonia that affect the function of the lungs. These findings are agreed with by other researchers [37, 38].

In our study, all COVID-19 suspected cases, based on clinical symptoms and laboratory findings, were tested by rRT-PCR to confirm the presence of viral infection. We reported that 6 patients showed false-negative results in the first rRT-PCR test that was confirmed in the next screening. rRT-PCR has proven its efficiency as fast, sensitive, and specific benchmark molecular diagnostic testing for the detection of several infectious agents, including viruses [28, 39, 40, 41].

Our findings regarding the WGS of SARS-CoV-2 showed that all the recovered strains belong to the GH clade. The validated sequences (n = 144) which recovered in the present study represented 60% of nucleotides sequences released from Egypt in 2020. Further analysis confirmed that all the Egyptian sequences were mutated and carried D614G, V5F, Q57H and G823S mutations. In agreement with this finding, several studies conducted to track the distribution of viral clades in different countries reported the higher prevalence of all clades with D614G mutation. Moreover, the spike proteins (SP) of all Egyptian tested strains (n = 144) are completely identical in that they have a G to D substitution at site 614 compared with the reference strain (NC_045512). All clades with D614G mutation are characterized by higher transmission, however, clades GH and GR showed higher virulence and significantly showed higher incidence among death or severe cases [42, 43, 44, 45, 46, 47, 48, 49].

Lamptey *et al.* reported that the D614G mutation was the most dominant mutation on the spike glycoprotein in about 84.2% of isolates in Africa, where it was detected in all isolates from Algeria, Morocco, and the Democratic Republic of Congo, and was co-occurred with E_V5F mutation in two Egyptian isolates. The D614G/E_V5F mutation was not observed in any other African country [50]. Furthermore, Showers *et al.* recorded that the Nsp12 thumb subdomain variant G823S was prevalent in samples collected during August, in Africa [51]. In the present study, we investigated 144 SARS-CoV-2sequences for detection of mutations landscape and confirmed that the most predominant subclade of D614G in Egypt was D614G/Q57H/V5F/G823S consistent with the results of Lamptey *et al.* who studied the epidemiology of SARS-CoV-2 in Africa and reported only one Egyptian sequence in which four mutations occurred together [50].

The constructed phylogenetic tree showed that the sequences of SARS-CoV-2 isolated in the current study were all identical and were closely related to Wuhan-Hu-1. Consistent with these findings are the results of a previous study that reported that whole-genome comparison of two Egyptian SARS-CoV-2 isolates (EPI_ISL_430819 and EPI_ISL_430820) revealed >99.8% identity with the reference isolate (NC_045512) [44].

Limited studies have been performed to trace the major SARS-CoV-2 genome haplotypes in the 2020 sequences and to identify new haplotype expansion globally [52, 53, 54]. The distribution of haplotypes over geographic regions is important for identifying the different clusters present throughout the world. Also, haplotype analysis is essential to understand the population-level divergence of SARS-CoV-2 and to identify the haplotypes associated with the reference strain (Wuhan-Hu-1) and reveal which ones have a high frequency of occurrence. In the present study, we reported the presence of 72 different haplotypes in 11 countries. The haplotype L.GL.YP.QT was frequently detected in all countries under investigation, while L.GL.YP.HI haplotype was the most prevalent globally but predominates in USA. However, all Egyptian sequences isolated in our study belong to L.GP.YP.HT haplotype and are similar to 23 Indian sequences and one French sequence.

## 4 Conclusion

All Egyptian SARS-CoV-2 isolates examined in the current study have at least 99% identity to the reference strain Wuhan-Hu-1. They were GH clade and L.GP.YP.HT haplotype. All Egyptian variants showed a unique mutation (D614G/Q57H/V5F/G823S) pattern. SARS-CoV-2 has evolved dramatically since its global spread and the clinical picture of the disease is still unclear. Therefore, our research is ongoing to isolate SARS-CoV-2 virus and perform genetic as well as clinical analysis to discover and follow its evolution in Egypt.

## 5 Materials and methods

### 5.1 Ethics statement

All subjects gave their informed consent for inclusion before they participated in the study. The study was conducted in accordance with the declaration of Helsinki, and the protocol was approved by Princess Nourah Bint Abdul Rahman, PUN Institutional Review Board (IRB Registration Number with KACST, KSA: H-01-R-059 and IRB Log Number: 20–0457) and the Research Ethics Committee of Faculty of Pharmacy, Tanta University. Samples collection was performed after permission from the head of the Army Hospital.

### 5.2 Sample collection

The samples were collected using oropharyngeal swabs from patients admitted to the Army Hospital from April 2020 to December 2020. All patients involved were suspected to be COVID-19 positive and showed possible symptoms of infection such as fever, cough, and difficulty of breathing. A total of 197 patients were selected for this study. The case definition of SARS-CoV-2 was according to the guidelines of the Ministry of Health and Population (MoHP) in Egypt [55]. For each patient, a full patient questionnaire was filled in and all recorded data were entered onto a Microsoft EXCEL spreadsheet. The designed patient questionnaire was comprehensive and covered demographics, clinical symptoms as well as laboratory test results.

Computed tomography scans were performed at the time of admission for all asymptomatic patients, and all screening results were consistent with COVID-19. Laboratory tests were performed and their results were collected, including results for complete blood count (CBC), C-reactive protein (CRP), prothrombin ratio (PT), international normalized ratio (INR), D-dimer, serum ferritin, liver function tests (alanine aminotransferase and aspartate aminotransferase), IL-6, creatinine, urea, and lactate dehydrogenase (LDH). Each Lab test was repeated four times with a time interval ranging from 4 to 5 days. The laboratory tests including CBC were performed using Swelab™ Alfa (Boule Diagnostics AB, Sweden). The coagulation tests including PT and INR were performed by KC1 Delta™ (Tcoag, Ireland). All the other tests were done using respons®920 (DiaSys, Germany). CT scan was performed by TOSHIBA Activion™ 16 CT Scanner (Toshiba Medical Systems Corporation, Japan).

### 5.3 Real-time reverse transcription polymerase chain reaction (rRT-PCR) test

The selected patients were confirmed to be SARS-CoV-2 positive by rRT-PCR. All oropharyngeal swabs were transferred on transportation media (MEM + antibiotics) at 4^○^C. For each sample, a volume of 140 μL was used for viral RNA extraction by QIAamp® Viral RNA Mini Kit (Qiagen, Germany) with internal PCR control. After purification of viral RNA, the next step was the preparation of the reaction mixture for PCR amplification. Real-time RT-PCR was performed using VIASURE® *SARS-CoV-2* Real-Time PCR Detection Kit (ref. VS-NCO212H) (Certest- Biotec S.L., Spain) in-order to semi-quantitatively amplify the extracted RNA using AriaMx Real-Time PCR system (Agilent, US) according to the manufacturer's guidelines.

### 5.4 Library preparation and next-generation sequencing

After total viral RNA extraction using QIAamp® Viral RNA Mini Kit, the AviSeq™ COV19 NGS Library prep kit from Avicenna™ (South Croydon, UK) was used for library preparation according to the manufacturer's guidelines. Whole genome sequencing was performed following the comprehensive workflow for detecting coronavirus using Illumina iSeq 100® System (Illumina, US).

### 5.5 Genome assembly

The DECOV™ online tool from Abiomix (https://abiomix.com/decov) was used to run accurate, fast, and automated NGS bioinformatics analysis of COVID-19 with minimal cost, time, and effort. Illumina raw reads were uploaded followed by quality control and filtering of the reads. After finishing the assembly, the de-novo assembly of each raw sequencing data was generated as FASTA file. Whole genome sequencing was performed on 184 isolated strains. The recovered validated sequences were submitted to NCBI Virus GenBank, given certain accession codes, and referenced by co-author Seadawy, M.G. In 2020, a total of 240 sequences were submitted NCBI Virus GenBank (https://www.ncbi.nlm.nih.gov/sars-cov-2/). (See Supplementary File S1).

### 5.6 Phylogenetic analysis

Phylogenetic trees are important to understand the emergence and evolution of SARS-CoV-2. All available SARS-CoV-2 sequences from Egypt (n = 240) till December 31, 2020, and Wuhan reference genome (NC_045512) were downloaded from NCBI Virus GenBank database. Bayesian analysis of molecular sequences was performed using BEAST/BEAGLE v1.10.4 software that uses Monte Carlo Markov Chains (MCMC) to average over tree space so that each tree is weighted proportional to its posterior probability [56]. TreeAnnotator v1.10.4 was used to summarize the maximum clade credibility (MCC) tree from the posterior distribution of trees and the MCC tree was visualized and edited in FigTree v.1.4.4.

### 5.7 Bioinformatics analysis

#### 5.7.1 Viral database development

The sequences of SARS-CoV-2 isolated in different countries were collected using National Center for Biotechnology Information (NCBI) SARS-CoV-2 Data Hub. Sequences were uploaded, processed, and analyzed in a temporary BIONUMERICS (v8.0) evaluation license, (bioMérieux, Applied Maths, Sint-Martens-Latem, Belgium) database with a SQLite backend. We have received permission from Applied Maths to publish the obtained results. Filtering of collected sequences was performed by comparing genome sequences to the NCBI reference strain, (Accession NC_045512). The sequences that showed high coverage (>29,000 bp) were selected. The *SarsCoV2* plugin components are listed in Table 5.

**Table 5:** The *SarsCoV2* plugin experiment components and their uses.

Component ID				Component type	Used for
SNP				**Character type**	The storage of the SNPs
SNP TRANSL				**Character type**	The storage of the translated SNPs
Genome				**Sequence type**	The storage of the (assembled) whole genome
ORF01_nsp01	ORF01_nsp02		ORF01_nsp03	**Sequence types (n = 26)**	The storage of the extracted subsequences
ORF01_nsp04	ORF01_nsp05		ORF01_nsp06		
ORF01_nsp07	ORF01_nsp08		ORF01_nsp09		
ORF01_nsp10	ORF01_nsp11		ORF01_nsp12		
ORF01_nsp13	ORF01_nsp14		ORF01_nsp15		
ORF01_nsp16	ORF02	ORF05	ORF03a		
ORF04	ORF06	ORF07a	ORF07b		
ORF08	ORF09	ORF10			

Genomic sequences can be uploaded into the database using the import routines available in BIONUMERICS either by downloading sequences from online repositories using their accession codes or by importing FASTA-formatted genomic sequences.

Imported genomic sequences were processed with the *SarsCoV2* plugin. The process includes the extraction of 26 subsequences from the genomic sequence stored in the genome experiment, saving these sequences in the corresponding destination experiments, and finally screening the 26 subsequences for SNPs.

#### 5.7.2 Clustering SNP data into dendrograms

The calculation engine BIONUMERICS was used for further interpretation of imported sequences via the WGS tools plugin. Each genomic sequence is separated (extracted) into subsequences, each of which is analyzed for SNPs relative to Wuhan-Hu-1. The Twenty-six subsequences were extracted from the imported genomic sequences and then stored in the corresponding destination experiments. The entries having incomplete SNP characters, with one or more missing subsequences, were excluded from the comparison. The dendrogram was calculated based on the complete linkage clustering algorithm and displayed in the "Dendrogram panel". A minimum spanning tree in BIONUMERICS was calculated in the "Advanced cluster analysis window".

#### 5.7.3 Haplotype determination

The *SarsCoV2* plugin allows determining haplotypes. The haplotype, as defined in the *SarsCoV2* plugin, is a set of high-frequency amino acid substitutions, organized by linkage groups.

### 5.8 Statistical analysis

Data were entered and analyzed using the Statistical Package for Social Sciences (SPSS) software version 26. Descriptive statistics were calculated to display demographic data and clinical symptoms in terms of mean and standard deviation for normal data and proportion, frequency, median, and IQR for non-normal data. The Chi-Square test was performed to examine the association between categorical variables that were presented as numbers and percentages. Mann-Whitney test was used to test the difference between non-normal numerical variables. Kolmogorov-Smirnov test was used for the normality tests. All P-values of <0.05 were considered statistically significant.

## 6 Institutional review board statement

All subjects gave their informed consent for inclusion before they participated in the study. The study was conducted in accordance with the declaration of Helsinki, and the protocol was approved by Princess Nourah Bint Abdul Rahman, PUN Institutional Review Board (IRB Registration Number with KACST, KSA: H-01-R-059 and IRB Log Number: 20–0457 in 2020) and the Research Ethics Committee of Faculty of Pharmacy, Tanta University. Samples collection was performed after permission from the head of the Army Hospital.

## 7 Informed consent statement

The purpose of the study was explained to the patients and informed consents were obtained according to the guidelines on human research adopted by the Research Ethics Committee at the Faculty of Pharmacy, Tanta University.

## Declarations

### Author contribution statement

Badriyah Alotaibi: Conceived and designed the experiments; Contributed reagents, materials, analysis tools or data.

Thanaa A. El-Masry: Conceived and designed the experiments; Contributed reagents, materials, analysis tools or data; Wrote the paper.

Mohamed G. Seadawy: Conceived and designed the experiments; Performed the experiments; Contributed reagents, materials, analysis tools or data.

Mahmoud H. Farghali, Asmaa Saleh and Yasmen ​F. Mahran: Analyzed and interpreted the data; Wrote the paper.

Bassem E. El-Harty and Mohamed S. Desoky: Performed the experiments.

Jackline S. Fahim and Mohamed M.E. Abd El-Monsef: Analyzed and interpreted the data.

Maisra M. El-Bouseary: Conceived and designed the experiments; Contributed reagents, materials, analysis tools or data; ​Analyzed and interpreted the data; Wrote the paper.

### Funding statement

This work was supported by Deputyship for Research & Innovation, Ministry of Education, Saudi Arabia (PNU-DRI-Targeted-20-(032).

### Data availability statement

Data included in article/supp. material/referenced in article.

### Declaration of interests statement

The authors declare no conflict of interest.

### Additional information

No additional information is available for this paper.

## Appendix A Supplementary data

Supplementary_Materials Supplementary File S1: List of the 240 genome sequences; their paper IDs - Accession codes - Release dates and Haplotypes; Supplementary File S2: List of the 18049 genome sequences imported to BIONUMERICS version 8.0 plugin; their paper IDs and haplotypes; Supplementary File S3: The results of CoVsurver Mutation Analysis of a selected isolate S143 (Accession Code: MW010028); Supplementary File S4: High-resolution plot of a dendrogram for Egyptian isolates (n = 240) and Supplementary File S5: High-resolution plot of Minimum spanning tree (MST) for all SARS-CoV-2 genomes (n = 18049) included in the present study. Supplementary_Materials
